# Biodegradable and Biobased Mulch Films: Highly Stretchable PLA Composites with Different Industrial Vegetable Waste

**DOI:** 10.1021/acsami.2c10965

**Published:** 2022-10-05

**Authors:** Danila Merino, Arkadiusz Zych, Athanassia Athanassiou

**Affiliations:** Smart Materials Group, Istituto Italiano di Tecnologia, Via Morego, 30, Genoa16163, Italy

**Keywords:** spinach stems, tomato pomace, cocoa shells, epoxidized soybean oil methyl ester, sustainable agriculture

## Abstract

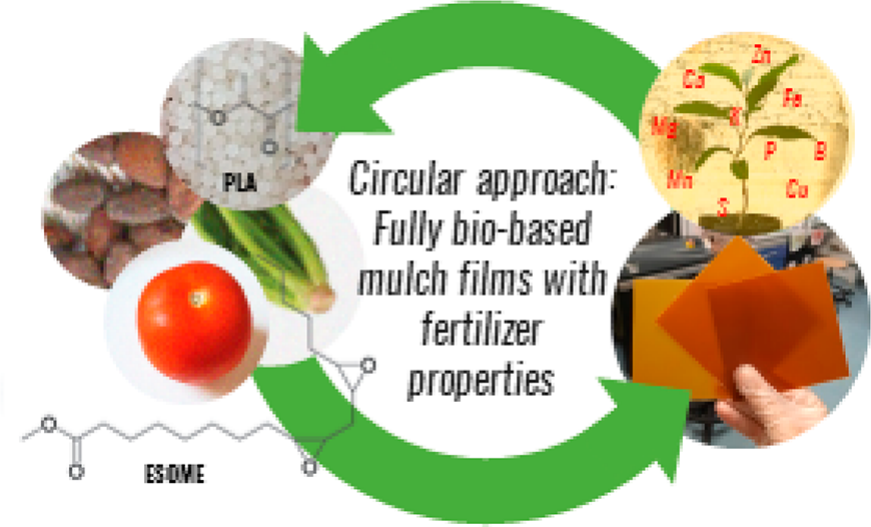

Highly stretchable biobased and biodegradable agricultural mulch films based on polylactic acid (PLA) and 10, 20, or 30 wt % various nonedible vegetable wastes such as spinach stems (SS), tomato pomace (TP), and cocoa shells (CS) are prepared and characterized in this work. The results demonstrate that appropriate PLA plasticization and vegetable waste addition allow for obtaining films suitable for mulching with tensile strengths in the 10–24 MPa range and elongations at break up to 460%, depending on the kind and amount of vegetable waste incorporated. Additionally, the developed mulches show low water solubility (1–15 wt %) and moisture content (1–3 wt %) with a water vapor permeability of up to 3 × 10^–10^ g s^–1^ m^–1^ Pa^–1^, similar to that of Mater-Bi. In addition, the type of vegetable waste added as filler were demonstrated to significantly affect not only the films’ mentioned properties but also their biodegradability. For instance, films prepared with 20 wt % SS were demonstrated to improve PLA soil biodegradability, which increased from 0 to 38 wt % for PLA composites after 6 months of a soil burial experiment. Lastly, the developed composites contain different amounts of plant micro- and macronutrients, indicating their potential as fertilizers. The results found in this work represent a sustainable, fully biobased alternative to other mulches already in the market.

## 1 Introduction

Agro-food systems have undergone significant transformations in the last century and the introduction of plastics in agriculture was the catalyzer for many of them.^1^ For instance, the use of mulch films, which are generally made of low-density polyethylene (LDPE), have allowed higher food yields with fewer resources thanks to the conservation of water in soil, the increase in the soil temperature, and the prevention of the growth of weeds. However, once the harvest is finished, they must be collected and properly discarded, a highly laborious and expensive task for producers.^1,2^ Instead, cultivators often opt for alternative methods that damage the environment and the health of the people living in the neighboring environment, such as incorporating mulches into the ground, burning them in the open air or disposing them of in local landfills. In addition, the use of petroleum-derived plastic agricultural mulches has been recently associated with the biological degradation of soil organic matter, the generation of greenhouse gases, and the environmental contamination with micro- and nanoplastics.^3−7^ Therefore, this food system needs to be renewed. The current need of increasing food productivity has to match the mitigation of the climate crisis and the transition to a circular economy, in accordance with the sustainable development goals of the United Nations.^2,8,9^

In this context, and considering the benefits of mulching, many researchers are working on the development of biodegradable mulch films (BDMs) suitable for replacing LDPE. The aim is to obtain BDMs that can be integrated into the soil during tillage and contribute to its enrichment in organic matter and micronutrients, when possible, after biodegradation.^7^ In this regard, numerous alternatives have been proposed and some materials are currently on the market. For example, BDMs based on polyesters such as poly(butylene adipate-*co*-terephthalate) (PBAT), known as Ecoflex, or polyesters combined with thermoplastic starch (TPS), known as and Mater-Bi, are commercialized by companies such as BASF and Novamont, respectively.^10−12^ These materials are biodegradable, but their fossil fuel-origin leads to the search of different materials of renewable origin that totally follow the circular economy concept. For this reason, different polysaccharides^13−16^ and proteins^17,18^ have been proposed as the basis for the preparation of entirely biobased agricultural mulches with rapid biodegradation in soil. Even bioplastics derived from the direct transformation of plant residues have been proposed for this application.^19,20^ However, their properties are very different from those of LDPE, which has caused skepticism in users and has prevented their introduction in the market. Therefore, the purpose of this research is to develop new biodegradable and fully biobased agricultural mulch films with similar properties to LDPE and at a comparable price.

Polylactic acid (PLA) is a biobased and biodegradable polyester, whose monomer, lactic acid, is obtained from the fermentation of starch or sugar. Its global market is constantly growing, with approximately 460 000 tons production in 2021.^21^ This polymer stands out for its good processability, mechanical properties, hydrophobicity, durability, and transparency. However, for its application as agricultural mulch, PLA has three main disadvantages: its low ductility, high price relatively to LDPE, and slow biodegradability in soil. PLA has an elongation at break of approximately 5%, is at least two times more expensive than LDPE and only degrades rapidly in industrial composting conditions at temperatures close to 58 °C.^22,23^

Recently, in our research group, a formulation that allows obtaining flexible PLA with a high elongation at break has been developed, overcoming the first of the previously mentioned drawbacks.^24^ For this, amorphous grade PLA was plasticized with epoxidized soybean oil methyl ester (ESOME) of renewable origin, reaching elongations at break of up to 800% depending on the content of incorporated vegetable oil. In this way, the plasticized PLA acting as mulch film could be applied in the field with the machinery currently used for the placement of the LDPE and could offer good mechanical resistance and integrity, as well as the prevention of the growth of weeds during the cultivation time.

The objective of this work is to obtain composite materials for biodegradable mulches, from the combination of amorphous PLA with 10 wt % ESOME, referred to as plasticized PLA (PPLA), and inedible vegetable residues as fillers at different concentrations. The vegetable wastes studied were spinach stems (SS), tomato pomace (TP), and cocoa shell (CS) powders. These residues were selected to evaluate whether different kinds of vegetable waste can affect PLA composite properties significantly. This is important not only to build knowledge but also to establish a stream of vegetable waste recovery from agro-food industry for their incorporation in the production chain of new value-added products such as mulch films. In the past, cellulose extracted from biomass was used in plasticized PLA composites as reinforcing agents.^25−27^ Here, we use the entire biomass in order to obtain not only a reinforcement effect but also a significant reduction in the price of the final materials, an incorporation of micro and macronutrients, and possibly, an improvement in PLA biodegradability in soil.

## 2 Materials and Methods

### 2.1 Materials

Amorphous PLA with a melt index of 8–10 g/10 min (210 °C) commercialized under the name of Ingeo biopolymer 6060D grade was purchased from NatureWorks (Minnetonka, MN, USA). Epoxidized soybean oil methyl ester (ESOME) was kindly donated by ATP R&D srl (Camisano Vicentino, VI, Italy) and was used as received. SS was kindly supplied by IDA S.r.l. (Alessandria, Italy), a company specializing in the commercialization of dried vegetables for the food industry, TP was provided by Conservas Martinete S.A. (Puebla de la Calzada, Badajoz, Spain), obtained as a byproduct of the preparation of tomato sauces, and CS by Ferrero S.p.A. (Piedmont, Italy), similarly obtained as a byproduct of the production of chocolates. SS have a chemical composition rich in cellulose and pectin, both at 35%,^19^ tomato pomace contains about 20% of cutin, a polyester made up of polyhydroxylated C16 and C18 fatty acids,^28^ and CS is a lignocellulosic residue that has 26% lignin and 24% cellulose.^29^

### 2.2 PLA and Industrial Vegetable Waste (IVW) Pretreatment

PLA pellets were ground into powder using a dry mill IKA Pilotina MC (Staufen, 

Germany) with a 3 mm sieve and dried under vacuum at 40 °C for 24 h. The vegetable residues were received dehydrated, but they were redried in an oven at 40 °C for 24 h to remove moisture residues. After that, they were ground in an Oster Versa 1400 blender and sieved using Endecotts Sieve Shaker Minor 200 (London, UK). The fraction of particle size inferior to 50 μm was collected and vacuum-dried at the same conditions as PLA, 40 °C for 24 h before extrusion, as indicated in the PLA data sheet. In order to prevent moisture regain, the PLA and the IVW were processed by extrusion directly after drying. Vegetable waste treated in these conditions presented a moisture content of 2 ± 1%. The morphology of the particles, analyzed by SEM, is included in Figure S1.

### 2.3 PLA-IVW Composite Preparation

After drying, ground PLA pellets were mixed with 10 wt % of ESOME (with respect to PLA) and 10, 20, and 30 wt % of IVW powder (with respect to the total material) in a glass beaker. The percentage of ESOME was selected at 10 wt % because the developed composites present appropriate mechanical properties and reduced migration of plasticizer at this concentration, while higher values lead to phase separation.^24^ The mixture was fed manually into a SCAMEX (Crosne, France) twin-screw extruder with 32 mm length and 18 mm diameter (L/D ratio of 1.7), under a nitrogen blanket, at 50 rpm, with a temperature profile of 110, 120, 130, 140, and 140 °C starting from the feeding section. Extruded filaments were pelletized and stored in polyethylene bags until hot-pressed. As a reference, pure PLA and plasticized PLA with 10 wt % ESOME (referred as PPLA) samples were also extruded under the same conditions. Films with an area of 100 cm^2^ and thickness of 500 μm (7 g) and 50 μm (2 g) controlled with the aid of a metal and thin sheet-Teflon molds, respectively, were prepared from pellets by compression molding at 140 °C using CARVER 4122 hydraulic press equipped with water cooling, using 5 min warmup time with no pressure applied followed by 5 min under 5 tons of pressure. The resulting films were labeled as summarized in Table 1.

**Table 1:** Sample Name and Composition

sample name	PPLA^a^ (wt %)	SS (wt %)	TP (wt %)	CS (wt %)
PPLA+10SS	90	10		
PPLA+20SS	80	20		
PPLA+30SS	70	30		
PPLA+10TP	90		10	
PPLA+20TP	80		20	
PPLA+30TP	70		30	
PPLA+10CS	90			10
PPLA+20CS	80			20
PPLA+30CS	70			30

a PPLA is the plasticized PLA with 10 wt % ESOME.

### 2.4 Characterization of PLA-IVW Composites

#### 2.4.1 Fourier Transform Infrared Spectroscopy (FTIR)

The chemical properties of films were investigated by FTIR in a VERTEX 70v equipment working under vacuum (<1 hPa) and using the attenuated total reflectance (ATR) with diamond crystal accessory. The spectra acquisition was carried out with the OPUS software at a resolution of 4 cm^–1^ and was the result of 64 scans. The band at 2995 cm^–1^ corresponding to the stretching vibration of C–H in CH_3_ groups was used to normalize the spectra.

#### 2.4.2 Scanning Electron Microscopy (SEM)

The cross-section morphology of films was investigated by scanning electron microscopy (SEM) in a JEOL JSM-6490LA microscope using the secondary electrons detector. For that, the samples were immersed in liquid nitrogen and then fractured. Cut samples were attached to aluminum stubs by using carbon tape in order to expose their cross-section surface and were covered with 10 nm of gold by sputtering. The micrographs were acquired with 10 kV accelerating voltage, load current of 78 μA and a magnification of 1000×.

#### 2.4.3 Mechanical Properties

Mechanical properties were analyzed by means of uniaxial tensile tests in an INSTRON 3365 machine. Before the analysis, the samples were conditioned in an environmental chamber (Espec SH-262, United States of America) at 24 °C and 50% RH for 48 h and cut to a dog-bone shape. The dimensions in the straight region of the bone were 25.01 mm in length and 3.98 mm in width. After that, the thickness of each specimen was measured with a digital micrometer (Mitutoyo, United States of America) with 0.001 mm accuracy. The drawing speed during the experiment was set at 5 mm min^–1^. At least five specimens of each sample were analyzed and their Young’s modulus (MPa), tensile strength (MPa) and elongation at break (%) were obtained. After a statistical analysis by one-way ANOVA, the results were presented as mean ± standard deviation (SD).

#### 2.4.4 Thermogravimetric Analysis (TGA)

Thermogravimetric analysis was carried out on a TA Q500 instrument in the thermal range from 30 to 600 °C under a N_2_ atmosphere (50 mL min^–1^) and a constant heating rate (10 °C min^–1^).

#### 2.4.5 Differential Scanning Calorimetry (DSC)

Differential scanning calorimetry was conducted in a TA Instruments Discovery DSC 250 at 10 °C min^–1^ heating rate and under nitrogen flow (50 mL min^–1^). Samples were heated from 0 to 100 °C, cooled down to 0 °C and heated again to 100 °C in order to remove any trace of moisture. Glass transition temperatures were determined using the TA TRIOS software at the midpoint of the change in the heat flow.

#### 2.4.6 Moisture Content (MC) and Water Solubility (WS)

Moisture content was determined gravimetrically. The initial and final weight (*W*_0_ and *W*_f_, respectively) of the analyzed samples were registered before and after drying in a vacuum oven at 40 °C for 24 h. MC was calculated following eq 1.
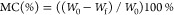
1Water solubility was instead calculated using eq 2. For that, dried samples of weight, *W*_d_, were put in contact with 5 mL of water for 24 h. After that, the water was removed and the films were dried again in a vacuum over at 40 °C for 24 h and their final weight, *W*_f_, was registered.
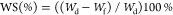
2Samples were analyzed in duplicates and results were reported as average ± standard deviation (SD). Statistical analysis by ANOVA and Tukey’s test was also performed.

#### 2.4.7 Water Vapor Permeability (WVP)

WVP was measured through a 100/0% RH gradient. For that, aluminum-based permeation capsules were filled with 300 μL of Milli-Q water (100% RH) and sealed on the top with the samples using two O-rings and a ring-shaped lid adjusted with screws. Test capsules were stored in a chamber at 0% RH simulated with dried silica gel. The capsules weight was monitored for 5 h. The slope obtained from the capsules weight loss (g) vs time (s) plot was divided by the exposed film area (m^2^) for the determination of the water vapor transmission rate (WVTR) and it was used in eq 3 for the determination of the WVP (g m^–1^ s^–1^ Pa^–1^):
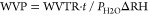
3where *t* is the average thickness of each sample (m), *P*_H__2O_ (Pa) is the vapor pressure of water at saturation and at the test temperature (20 °C), and ΔRH is the difference in vapor pressure through the film. All samples were analyzed in triplicates and results were expressed as average ± SD. Statistical analysis by ANOVA and Tukey’s test was also performed.

#### 2.4.8 Optical Properties

The direct transmittance (%) of light was measured in a Cary 6000i UV–vis–NIR spectrophotometer in the 400–700 nm range, which is the photosynthetically active radiation (PAR) range. The direct transmissivity coefficients, τ_PAR_^Direct^, were then calculated as average values of the spectral transmissivity, τ(λ), over the PAR range considering the spectral distribution of the solar radiation at the Earth’s surface,^30^*S*_*λ*_, as a weighting function as shown in eq 4:
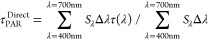
4where Δλ was equal to 10 nm and is the wavelength interval used in the calculation. All the curves were normalized to a thickness of 100 μm to allow samples comparison.

#### 2.4.9 Price Estimation

The cost of the mulch films presented in this work was estimated considering only the cost of raw materials. The price of vegetable waste is assumed to be 0 $/kg. The ESOME price of 0.095 €/kg was taken from the GUIDECHEM Web site^31^ and the price of PLA (2.14 €/kg) was taken from the work published by Hann et al.^23^ As a reference, the prices of LDPE (0.95 €/kg)^32^ and two biopolymers commonly used for mulch films, Mater-Bi (2.76 €/kg)^33^ and PBAT (4.57 €/kg),^34^ were taken from the Alibaba website.

#### 2.4.10 In-Soil Biodegradation Assay

The biodegradability of selected films and their respective controls (PLA and PPLA) was analyzed over a 6 month-experiment following the methodology reported by Merino et al.^35^ Samples were cut to the 3 cm × 3 cm dimension, put into a PE-mesh bag and buried in the biodegradation media. For that, a pot of 25 cm × 25 cm x 16 cm was filled with the soil collected from the proximity of the Istituto Italiano di Tecnologia located in the Morego hill in the outskirts of Genoa (Italy). The soil is classified as Dystric Cambisol by the World Reference Base for Soil Resources (WRB) and has a 24.7% of clay and a cation exchange capacity of 9.32 cmol/kg.^36^

The water content was measured gravimetrically after drying a soil sample at 105 °C until constant weight. The final soil moisture was adjusted to 16% (50% of the water holding capacity) and the final soil pH was 7.0, measured in a mixture of 50 g of soil and 100 mL of tap water.

The assay was conducted indoors. The average temperature was 20 ± 2 °C and the average relative humidity was 60 ± 5% RH. Samples were initially dried for 24 h at 40 °C in a vacuum oven and weighted (*W*_0_). Then, they were placed in handmade PE-mesh bags and buried in the soil. The samples were removed at the months 1, 3, and 6, and the soil attached to the samples was carefully removed with a brush. Unburied samples were dried overnight in a vacuum oven at 40 °C and reweighted (*W*_*t*_). Finally, the weight loss (%) of each sample was determined as shown in eq 5. Results are represented as a function of time (months).
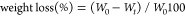
5Samples were analyzed in duplicates and results were expressed as average ± SD. Pictures and SEM micrographs of dried films were also taken.

#### 2.4.11 Gel Permeation Chromatography (GPC)

Gel permeation chromatography (GPC) was performed using an integrated OMNISEC system (Malvern Panalytical Ltd., UK) equipped with a D6000 M and T4000 columns (10 and 7 μm particle size, respectively, 300 × 8 mm) and a triple detection method (refractive index, a viscometer and a dual angle light scattering detector at 15° and 90°). Prior to each analysis, samples were dissolved in THF at room temperature and the solution was filtered through a 0.22 μm PTFE filter. THF stabilized with 250 ppm BHT was used as an eluent at a temperature of 35 °C and a flow rate of 0.8 mL/min. The system was calibrated with polystyrene (PolyCal standards, Malvern Panalytical Ltd., UK) 105 kDa narrow standard of known dispersity, intrinsic viscosity and d*n*/d*c*. Data analysis was performed using *OMNISEC* software V11.32 (Figures S4–S10). Since the software was not able to properly calculate the molecular weight of the samples containing SS due to the problems with the baseline of the dual angle light scattering detector (Figures S8 and S9), the molecular weights of all the samples were calculated manualy (Table S2) using the refractive index detector and the relationship between the retention volume and the molecular weight established for pure PLA (Figure S10). Further in this publication only those molecular weight values will be used. Number average molecular weight (*M*_n_), weight average molecular weight (*M*_w_) and dispersity (*Đ*) were calculated using the following equations, where *N*_*i*_ is the number of molecules with weight *M*_*i*_:
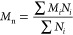
6
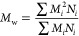
7
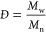
8

#### 2.4.12 Inductively Coupled Plasma (ICP)

The quantification of the micro- and macro-nutrients present in PPLA composites with 20 wt % of SS, TP and CS was performed using an inductively coupled plasma-atomic emission spectrometer (ICP-AES, iCAP 7600 DUO, Thermo, Bremen, Germany) after overnight digestion of the samples in aqua regia.

#### 2.4.13 Statistical Analysis

Results were reported as mean ± SD. One-way analysis of variance (ANOVA) and Tukey’s test were applied to determine if significant differences were found among mean values at a 0.05 level of significance using the *Origin* 2019b software.

## 3 Results and Discussion

### 3.1 Chemical and Morphological Characterization

Chemical interactions among PLA-based mulch film components were investigated by FTIR, and morphological changes in their bulk microstructure after plasticization and vegetable waste powder addition were followed by SEM.

**Figure 1:**
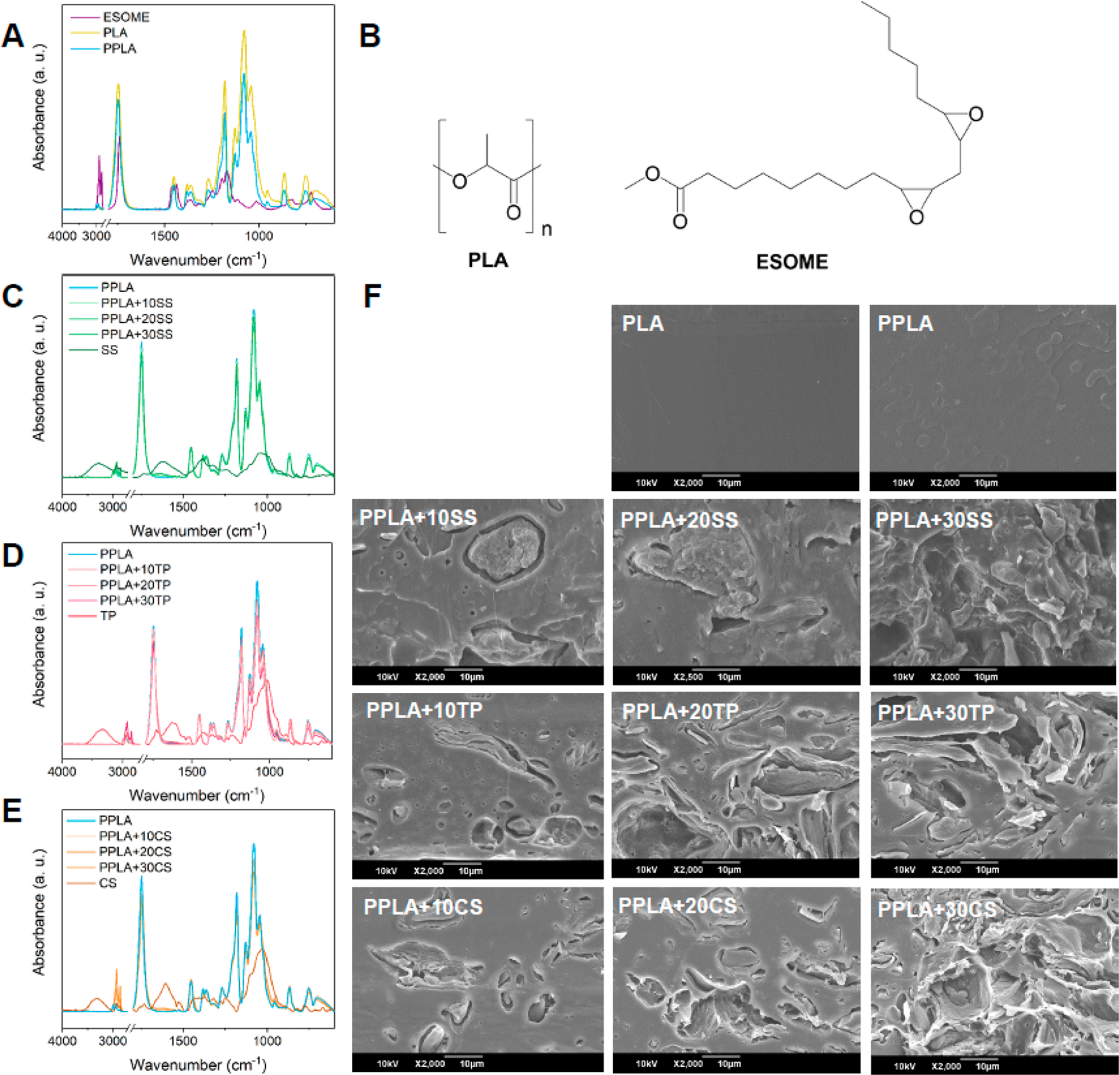
Results of the chemical and morphological characterization of PLA-based mulch films. (A) FTIR spectra of amorphous PLA, plasticized PLA (PPLA), and the plasticizer ESOME. (B) Chemical structure of PLA and ESOME. (C) FTIR spectra of PPLA, raw SS powder, and PPLA composites with 10, 20, and 30 wt % SS. (D) FTIR spectra of PPLA, raw TP powder, and PPLA composites with 10, 20, and 30 wt % of TP. (E) FTIR spectra of PPLA, raw CS powder, and PPLA composites with 10, 20, and 30 wt % CS. (F) SEM micrographs of the freeze-fractured surface of PLA, PPLA, and its composites with 10, 20, or 30 wt % SS, TP, or CS.

Figure 1A shows the infrared spectra of PLA, ESOME, and PPLA. PLA presents the characteristic bands associated with the C–H stretching (3000–2850 cm^–1^) and bending (1450 cm^–1^) frequencies of CH_3_ groups, and the carbonyl stretching (1746 m^–1^) and bending modes (1267 cm^–1^) characteristics of ester groups (Figure 1B).^37^ On the other hand, ESOME presented similar chemical bonds, but its peaks appeared at slightly different position together with a new peak centered at 825 cm^–1^ (Table S1), associated with the presence of the epoxide group, absent in PLA (Figure 1B).

After the incorporation of ESOME into the PLA matrix, small changes were observed in the position of the previously mentioned stretching and deformation bands (Table S1), suggesting hydrophobic interactions among components.^38^ In addition, it was observed that the intensity of the PLA band absorption decreased upon ESOME addition, according to its reduced content in the final formulation, and the stretching band indicating the presence of epoxide groups was no longer visible (Figure 1A). This last observation may be due to the low ESOME content in the formulation, as demonstrated by Zych et al.,^24^ who studied the PPLA films by ^1^H NMR and confirmed the presence of the epoxide, contrary to the theory of other authors considering that ESOME could have reacted with terminal OH groups in PLA.^39,40^

Subsequently, with the addition of different percentages of vegetable waste to the formulation, some changes were observed (Figures 1C–E and Table S1). The peaks related to the C–H stretching (3000–2800 cm^–1^) increased their intensity, broadened, and were slightly displaced. Besides, a broad band around 1696–1540 cm^–1^ appeared, which is associated with the presence of aromatic groups (C=C–C stretching) and amides (N–H deformation and C–N stretching), which are part of a broad number of phytochemicals,^41,42^ as seen in the FTIR spectrum of the raw SS, TP, and CS powders also included in Figure 1C–E as references.

Figure 1F shows the PLA freeze-fractured surface, where a completely homogeneous and smooth surface can be observed with some small lines or striations typically associated with a brittle fracture. On the contrary, when ESOME is added, some irregular patterns are observed on the surface that correspond to a more ductile fracture.^43,44^ In addition, PPLA presented a homogeneous surface, which indicates that the plasticizer had a good integration in the matrix, in accordance with what was previously reported for this system.^24^ After the cryogenic fracture of the PPLA composites with SS, TP, and CS, the surface appeared rough and heterogeneous, and these characteristics become more intense with the content of incorporated vegetable residues. The different fillers were observed in the form of particles without a defined shape, weakly bound to PPLA, probably due to the poor chemical compatibility between the hydrophobic PPLA and the hydrophilic plant residues, rich in OH (3600–3000 cm^–1^), C–OH and C–O–C groups (1180–950 cm^–1^), as shown in the FTIR spectra of Figures 1C-E.

#### Thermomechanical Properties

**Figure 2:**
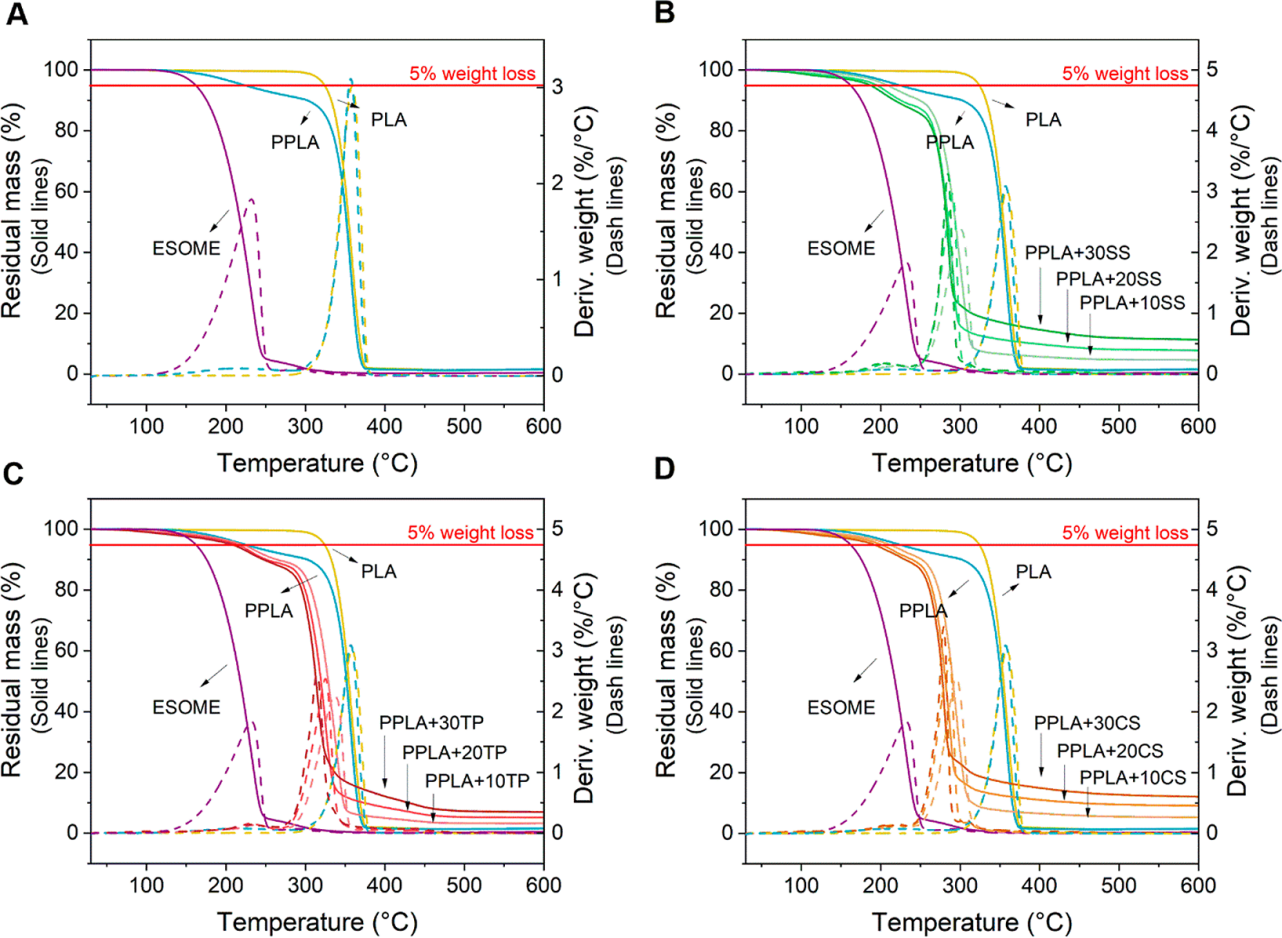
Thermogravimetric analysis of the PLA-based mulch films. (A) TGA and DTGA curves for PLA, PPLA, and ESOME. (B) TGA and DTGA curves for PLA, PPLA, ESOME, and PPLA composites with 10, 20, and 30 wt % SS. (C) TGA and DTGA curves for PLA, PPLA, ESOME, and PPLA composites with 10, 20, and 30 wt % TP. (D) TGA and DTGA curves for PLA, PPLA, ESOME, and PPLA composites with 10, 20, and 30 wt % CS. In all cases, solid lines refer to the TGA curves and dashed lines to the DTGA curves. A red line in each plot is pointing to 5 wt % weight loss, so reduction in thermal stability can be easily noted.

Thermomechanical characterization of PPLA composites was done by TGA, DSC and tensile tests. TGA and derivative TGA (DTGA) curves for PLA, ESOME and PPLA are included in Figure 2A. This figure shows that both PLA and ESOME showed a single event with a maximum degradation rate at 359 and 232 °C, respectively. Once a 10 wt % ESOME was added to PLA to obtain PPLA, an intense peak was observed with a maximum at 357 °C, and another very small and wide peak was centered at 214 °C, corresponding to ∼10% weight loss, likely associated with the evaporation of the ESOME plasticizer. In addition, the use of ESOME as a plasticizer produced a 100 °C reduction in the thermal stability of the plasticized film, considering that PLA and PPLA lose 5 wt % at 323 and 232 °C, respectively (Table 2).

**Table 2:** Results of Thermal Analysis of PLA-Based Mulch Films by TGA and DSC

	temperature at 1% mass loss (°C)	temperature at 5% mass loss (°C)	temperature at maximum degradation rate (1st event, °C)	temperature at maximum degradation rate (2nd event, °C)	*T*_g_ (°C)
PLA	300.6	323.8		359.0	54.9
PPLA	160.1	223.1	214.2	357.1	29.8
PPLA+10SS	128.6	207.9	219.6	301.1	31.5
PPLA+20SS	98	194.7	208.2	290.5	31.7
PPLA+30SS	94	186.5	203.2	284.5	32.5
PPLA+10TP	122	219.8	238.2	337.2	31.2
PPLA+20TP	104	213.2	231.7	325.2	33.4
PPLA+30TP	102	206.7	230.7	314.5	35.1
PPLA+10CS	111	210.7	228.9	295.1	28.7
PPLA+20CS	96	199.8	218.9	286.7	30.0
PPLA+30CS	96	191.2	204.2	279.1	30.2

In this way, the maximum temperature selected for processing the materials by extrusion (140 °C) appears as an appropriate temperature to preserve the chemical structure of PLA and ESOME, as has been previously demonstrated.^24^ However, when the vegetable wastes were added, the thermal stability decreased a bit more (Table 2), and some extent of vegetable waste degradation can be expected.

When fillers are added, the composite films exhibited degradation in two stages. The first one, between 160 and 270 °C, is associated with the evaporation of the plasticizer and the thermal degradation of some compounds present in the fillers, such as pectin and hemicellulose polymers that, according to the literature, degrade partially overlapping in the range 190–300 °C.^19,45^ A second, much more significant weight loss event could be observed between 250 and 350 °C, due to the joint degradation of PPLA and other compounds present in SS, CS, and TP, such as cellulose and lignin polymers that, according to the literature, show maximum degradation rates between 300 and 380 °C.^19,45^

Furthermore, this degradation peak shifted to lower temperatures with increasing content of incorporated plant residues (Table 2). As can be seen from the first degradation stage (Table 2), the thermal stability of the composite films decreased when the vegetable residues were added and this decrease become subtly more significant with the incorporated vegetable residue content. Similar results were reported by other authors for mixtures of PLA and banana fiber,^46^ PLA, and silver skin (byproduct derived from the roasting of coffee beans),^47^ and PLA-cocoa bean shells composites prepared by casting.^48^ Interestingly, the PPLA-TP composites presented the highest thermal stability compared to the PPLA+SS and PPLA+CS composites, probably due to the presence of cutin, a polyester with a maximum degradation rate between 230 and 270 °C.^49^

Concerning DSC analysis, PLA-based mulch films presented only a single glass transition with no cold crystallization in the range of temperature analyzed (0–100 °C), as expected since amorphous PLA was used in this work. Typically, amorphous PLA shows a *T*_g_ value around 55–60 °C,^50^ similar to what was observed here. Once ESOME was added, the *T*_g_ value decreased significantly, according with the expected plasticizing effect that allows PLA chains to increase their mobility. Then, after incorporation of vegetable residues into PPLA, *T*_g_ values increased slightly when compared to PPLA, suggesting that they can act as reinforcements. Moreover, increasing the amount of vegetable waste incorporated lead to a slight increase in the *T*_g_ and among different fillers, TP produced the highest *T*_g_ increment.

These results were directly correlated with the results obtained for the mechanical properties of the PLA-based mulch films. PLA was tremendously plasticized by ESOME, which produced a decrease in the Young modulus of more than 50% and an increment in the elongation at break of about 10 000%, reducing TS values from 56.1 to 32.5 MPa (Figure S2), in agreement with the results previously reported for this system.^24^

**Figure 3:**
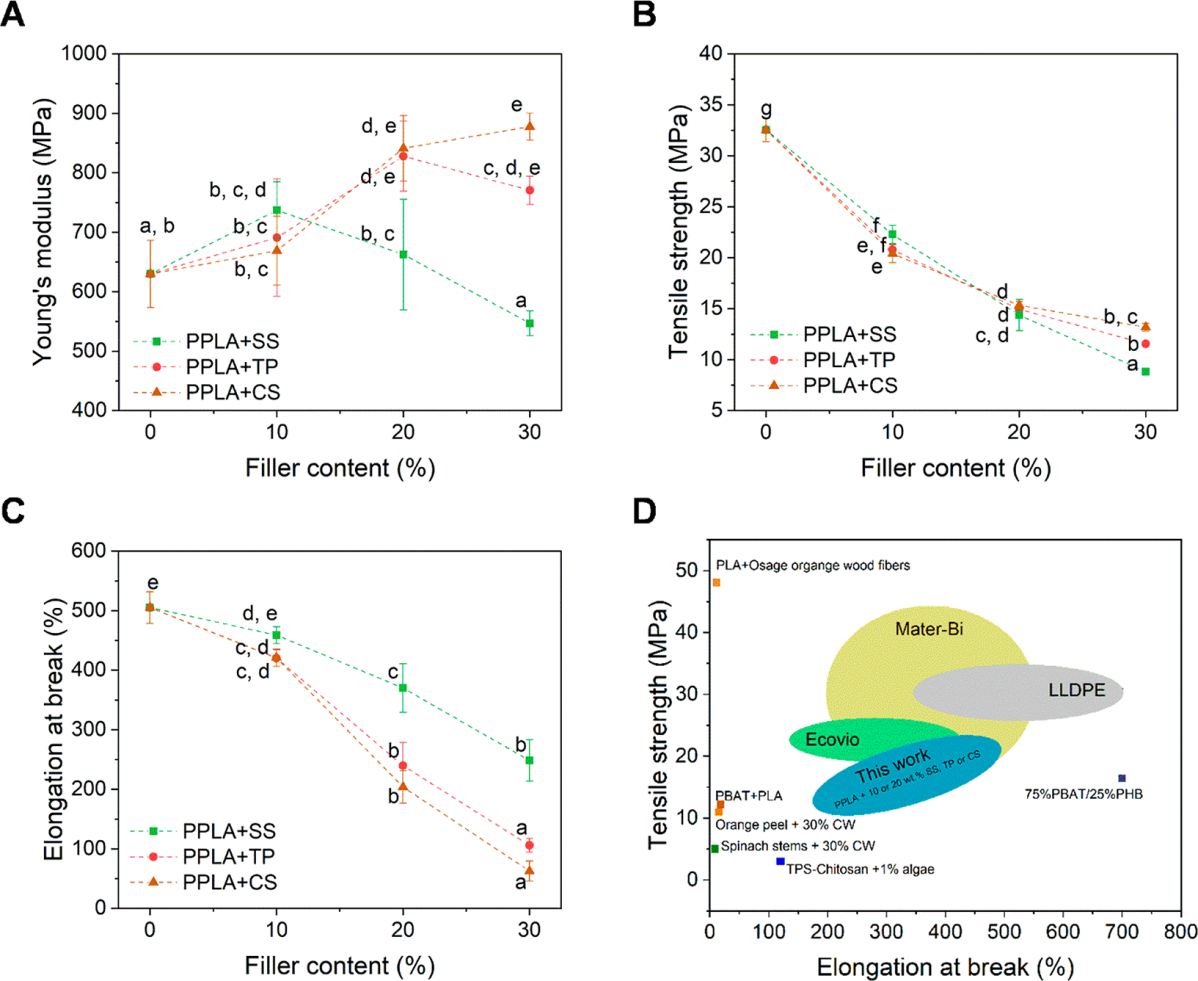
Mechanical properties of PLA-based mulch films and their comparison with other mulches’ properties found in the literature. (A) Young’s modulus (MPa) of PPLA and its composites with 10, 20, and 30 wt % SS, TP, or CS. (B) Tensile strength (MPa) of PPLA and its composites with 10, 20, and 30 wt % SS, TP, or CS. (C) Elongation at break (%) of PPLA and its composites with 10, 20, and 30 wt % SS, TP, or CS. (D) Comparison of the mechanical properties of the materials developed in this work (PPLA+10SS, TP, or CS and PPLA+20SS, TP, or CS) with those of commercial mulch films^11,51,52^ and others found in the literature.^13,19,53−55^

After adding the fillers, an antiplasticizing effect was observed for PPLA+TP and PPLA+CS composites with 20 and 30 wt % of TP or CS (Figures 3A–C). These materials presented a marked increase in the materials Young’s modulus, reaching about 139% increment when compared with PPLA (Figure 3A), accompanied by a significant reduction of material elongation at break, reaching an *E*_b_ of about 63% for PPLA+30CS (Figure 3C). Composites prepared with SS or with 10 wt % TP and CS did not show a significant increase in the elastic modulus, but they still show a decrease in their elongation at break when compared to PPLA (Figures 3A, C). Regarding TS, all the composites showed an exponential decrease with the increment in the filler content (Figure 3 B). This result can be attributed to the weak interaction between PPLA with SS, TP, and CS fillers as observed previously by FTIR and SEM.

Considering these results in the context of the mulching application, the materials prepared with 10 and 20 wt % vegetable waste presented the most suitable stiffness and stretchability. Figure 3D compares the mechanical properties of these two types of PLA composites with other composites reported in the literature for mulch production, such as those prepared from vegetable waste,^19^ PLA and Osage Orangewood fibers,^53^ PBAT-PHB blends,^55^ and TPS-chitosan blends with algal filler.^13^ In addition, Figure 3D also compares the materials developed in this work with those currently in the market. Interestingly, PPLA + 10 or 20 wt % SS, TP, or CS showed TS and *E*_b_ values similar to those of Mater-Bi and Ecovio, demonstrating great potentiality for their replacement.

#### Water Interaction

**Figure 4:**
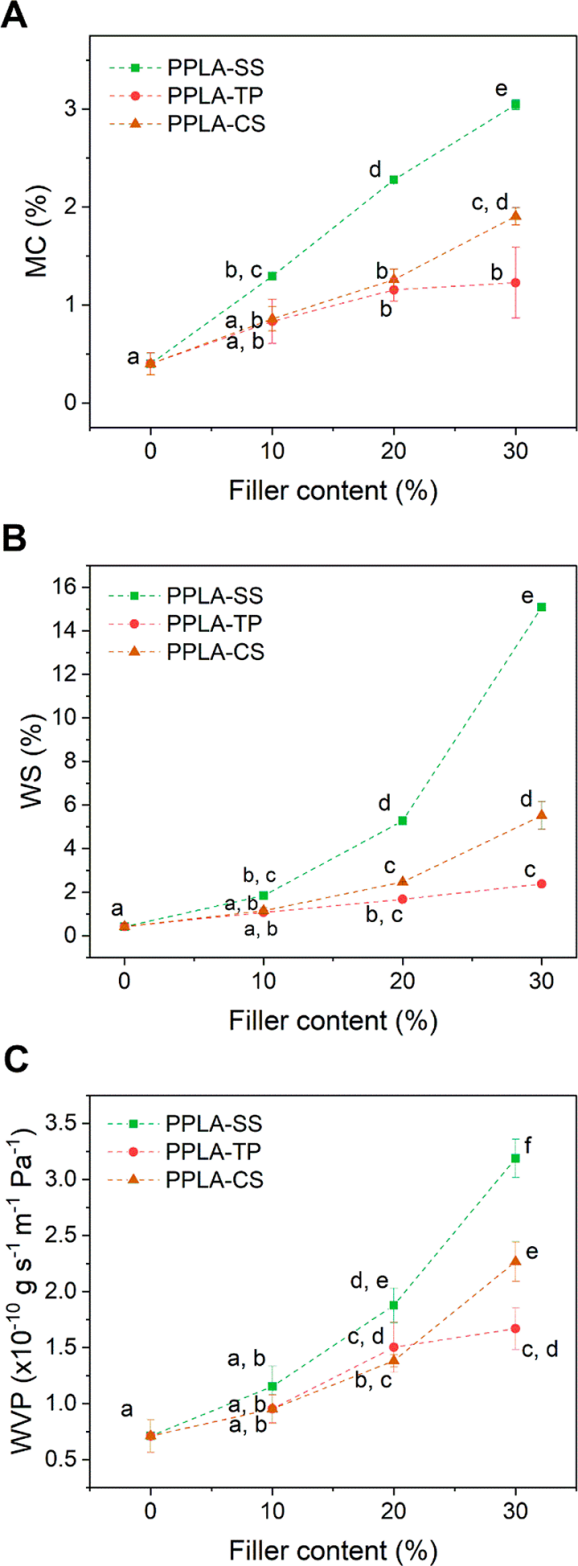
Properties related to the PPLA composites interaction with water as a function of the filler content. (A) Moisture content (MC), (B) water solubility (WS), and (C) water vapor permeability (WVP).

The behavior of PLA-based mulches upon interaction with water and humidity were investigated by determining their moisture content (MC), solubility in water, and permeability to water vapor. Ideally, mulch films should act as a physical barrier that reduces water evaporation from the soil while helping to avoid the need for frequent watering.^1^ In Figure 4A–C it can be seen that both the kind of filler and the amount added had a significant effect on PPLA composites properties. The filler significantly affected the interaction of these materials with water only when added at 20 or 30 wt %. From Figure 4A, it can be seen that the MC increased gradually with the addition of the fillers and that this increment was steeper for SS, with 3% MC being the highest value for 30 wt % SS into PPLA. Similarly, PLA-SS composites showed the highest WS (Figure 4B) and WVP (Figure 4C) when SS was added at 30 wt %. Instead, for fillers addition at 10–20 wt %, the increase in these properties was very small. For instance, MC increased from 0.4 to 1.2%, WS increased from 0.4 to 2.5% and WVP increased from 0.7 × 10^–10^ to 1.4 × 10^–10^ g s^–1^ m^–1^ Pa^–1^ when adding 20 wt % of CS to PPLA. The values obtained here for the WVP were similar to the ones reported by Mariniello et al.^56^ for Mater-Bi, 4.8 × 10^–10^ g s^–1^ m^–1^ Pa^–1^, demonstrating once again the great potential of the films for mulching.

#### Interaction with Light

The interaction of the materials with light is another critical parameter that can significantly affect the functionality of the mulches. Opaque to photosynthetically active radiation (PAR) mulches can obstruct the passage of the light through the films to the soil. Consequently, this kind of mulch film is used to prevent the growth of weeds. Besides, light interaction with the mulches has a further implication on the soil temperature. In particular, black mulches are used to increase the soil temperature, while white and silver mulches reflect the light and are used to lower soil temperature in cases where high temperatures can harm plants and reduce yields.^57^ Colored mulches also exist that have been reported to influence pest control and have specific effects on plant growth. For example, Shiukhy et al.^58^ have reported that red mulches increased strawberry fruit weight and quality when compared with black and white mulches, while the work of Greer et al.^59^ reviewed the beneficial effect of colored mulches on insect pests that vector viruses such as aphids, thrips, and whiteflies.

**Figure 5:**
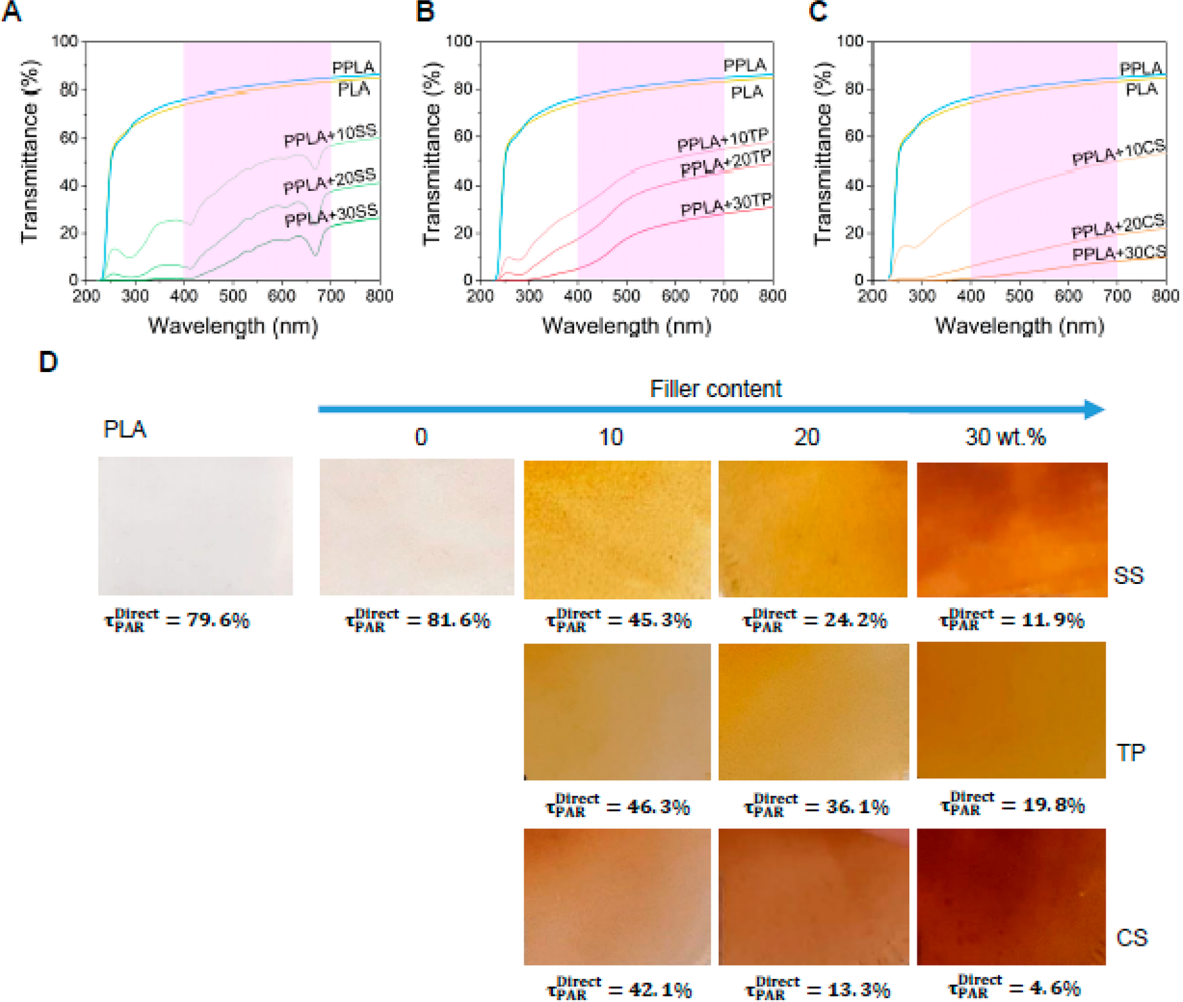
Optical properties of developed mulch films. (A) UV–vis spectra of PLA, PPLA, and the PPLA composites with 10, 20, and 30 wt % SS filler; (B) UV–vis spectra of PLA, PPLA, and the PPLA composites with 10, 20, and 30 wt % TP filler; and (C) UV–vis spectra of PLA, PPLA, and the PPLA composites with 10, 20, and 30 wt % CS filler. (D) Images of developed composites on the top of a white piece of paper for the visual evaluation of their transparency and values of the direct transmission coefficient in the PAR region. All the films photographed presented an average thickness of 70 μm.

In this work, the light transmission in the PAR range, 400–700 nm, decreased when the filler content increased (Figures 5A–C). The values of direct transmissivity in that range included in Figure 5D show that the optical properties also depend on the kind of vegetable waste used as filler. Films with 30 wt % filler were the most opaque, but they still show a coefficient above the black PE mulch films (0%).^60^ Therefore, these materials could be used as colored mulches and their effect on different crops should be tested. However, if they are intended to replace black mulch films, other strategies should be adopted to decrease the PAR light transmission. For example, the addition of biochar particles prepared directly from the pyrolysis of the vegetable wastes has been demonstrated to effectively lower the transmissivity coefficients below 3% with the addition of only 5 wt % filler, as previously reported by the authors.^20^

#### Economic Considerations

To assess the commercialization potential of the presented mulch films, we estimated their cost and compared it to LDPE and two biodegradable commercial polymers commonly used for mulch films: Mater-Bi and PBAT. PLA (2.14 €/kg)^23^ is cheaper than Mater-Bi (2.76 €/kg)^33^ and PBAT (4.57 €/kg),^34^ but it cannot compete with LDPE prices (0.95 €/kg).^32^Table 3 shows the values of the composites with the addition of different contents of vegetable waste. The 10 wt % addition of ESOME alone allows for a lower cost of 1.94 €/kg, down from 2.14 €/kg. Further addition of nonedible vegetable parts can lower the cost even more, making these composites a lot cheaper than other biodegradable alternatives on the market and much closer to the LDPE price.

**Table 3:** Estimation of PPLA Composite Price and Comparison with Commercial LDPE, Mater-Bi, and PBAT Pellet Prices

Material	Price (€/kg)	Refs
PLA	2.14	(23)
PPLA	1.94	this work
PPLA+10% vegetable waste^a^	1.74	this work
PPLA+20% vegetable waste^a^	1.63	this work
PPLA+30% vegetable waste^a^	1.55	this work
LDPE	0.95	(32)
Mater-Bi	2.66	(33)
PBAT	4.57	(34)

a Vegetable waste cost was considered 0 €/kg.

#### Biodegradability in Soil and Fertilizer Potential

**Figure 6:**
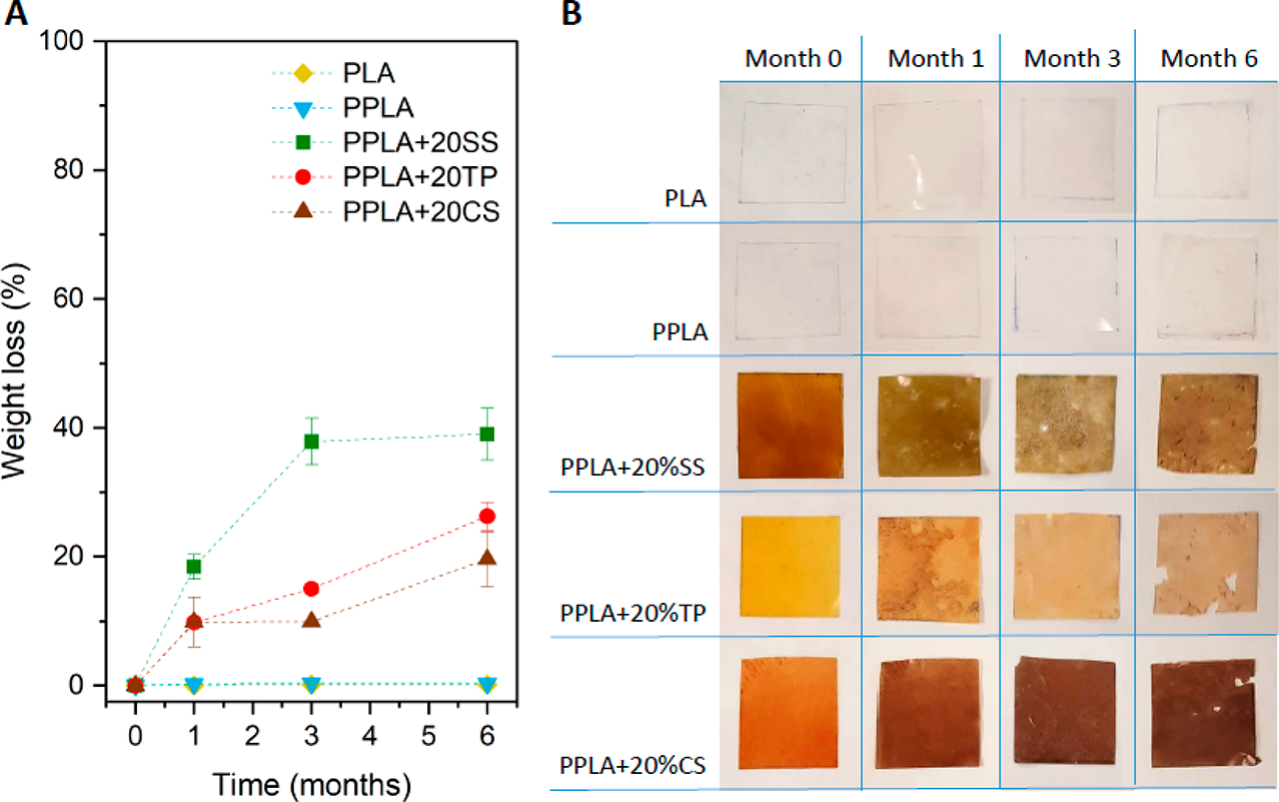
Results of PLA composites in soil-biodegradability. (A) Curves of weight loss (%) vs time (months). (B) Images of unburied film surfaces after 0, 1, 3, or 6 months of biodegradation assay.

Mulch films prepared from PPLA and 20 wt % SS, TP, and CS were selected for biodegradability tests because of their good mechanical, barrier, and optical performance and because they offer the lower price without significantly compromising the properties of mulches. PLA and PPLA films were used as controls during the experiment. The weight loss curves and the pictures of the films’ surfaces during the biodegradability test are included in Figures 6A, B, respectively. In addition, microscopic changes on the surfaces of films were investigated by SEM (Figure S3), and selected samples were analyzed for PLA molecular weight determination by gel permeation chromatography (Table S2).

The addition of IVW had a significant effect on the composite biodegradability. Even at one month of the soil burial test, PPLA composites showed a significant weight loss and started to show color changes likely caused by the microbial attack. These changes were exacerbated during the rest of the experiment. Bioplastics showed holes and rough surface morphology, while PLA and PPLA showed almost no weight loss and changes in surface morphology (Figure 6B and Figure S3). This increase in the roughness of the materials and color change could be due to the enzymes excreted by microorganisms capable of degrading blend components.^61^ Similar biodegradability behavior was observed by Siakeng et al. for PLA composites with coir and pineapple leaf fibers combined in different percentages, representing a total 30 wt % filler content.^62^ The authors of that work found a maximum of 18 wt % weight loss for the PLA composites after 5 months of soil burial. In this work, a higher biodegradability was observed, probably because of the filler’s chemical composition. In fact, the chemical composition of the fillers played a crucial role in the biodegradability of the PPLA composites. Fillers with a lignocellulosic composition presented the lowest values of weight loss, according with the slower biodegradation of lignin in comparison with polysaccharides^63^ (Figure 6A).

To determine if the IVW can boost PLA biodegradability, GPC measurements were conducted on PPLA+20SS samples before and after the 6-months soil burial experiment. Pure PLA and PPLA samples were also analyzed as controls. For all the samples, a small decrease in molecular weight was detected after 6 months of soil burial (Table S2), which might suggest the beginning of biodegradation. The results indicate that PLA biodegradability was slightly affected by the presence of the ESOME plasticizer or the filler with nonsignificant differences among these components (the reduction in the molecular weight was about 10 000 Da for the PPLA and PPLA+20SS after the 6 months). Therefore, the high weight loss observed in Figure 6A (around 38%) for the PPLA+20SS sample might be caused by the combined fast biodegradation of SS and ESOME plasticizer with erosion of the sample and subsequent disintegration to small fragments that cannot be collected from the soil.

Lastly, the mineral composition of the PPLA composites with 20 wt % SS, TP, and CS was determined by ICP. Results included in Table 4 demonstrate that the materials developed can provide micro- and macronutrients to the soil after the biodegradation of the films. This result is significant because it can contribute to the prevention of excessive use of fertilizers, which are associated with several pollution issues,^2^ and at the same time demonstrates one extra benefit of incorporating vegetable wastes into the production chain of new added-value materials, especially in agriculture.

**Table 4:** Micro- and Macronutrient Content in PPLA Composites^a^

	Mineral composition (ppm)									
Sample	B	Ca	P	Cu	Fe	K	Mn	Mg	S	Zn
PPLA+20SS	0.03	5.81	0.96	0.04	0.05	24.43	0.03	5.00	0.58	0.02
PPLA+20TP	0.02	3.58	0.30	0.01	0.02	4.13	0.04	2.27	0.31	0.01
PPLA+20CS	0.02	4.49	0.01	0.02	0.01	26.54	0.01	1.78	0	0

a The method used for mineral quantification was not able to detect the N element.

## Conclusions

Amorphous PLA was successfully plasticized and combined with 10, 20, or 30 wt % different industrial vegetable wastes (spinach stems, tomato pomace, and cocoa shells) by melt extrusion, followed by compression molding. The properties of the materials obtained were shown to be dependent on the filler content and type. Composites with 10 or 20 wt % filler showed potential for mulching based on their mechanical, water vapor barrier, and optical properties, similar to commercial BDMs, while the materials with 30 wt % filler presented drastic deterioration in their mechanical properties, in water absorption, and in thermal resistance. Although the prices of PPLA composites with vegetable waste are higher to those of LDPE films, the addition of fillers significantly affected their biodegradability, reaching almost 40 wt % biodegradation after 6 months when using 20 wt % SS. The materials developed also showed a wide variety of micro- and macronutrients, beneficial for plant growth and development. Future work will test the performance of these materials in real agricultural conditions.

## Supporting Information Available

Infrared spectroscopy for PLA, ESOME, PPLA, and their composites with SS, TP, and CS; mechanical properties of PLA and PPLA; SEM micrographs over the biodegradability test; molecular weights by GPC (PDF)
