# Supplementary material for: Biodegradable and Biobased Mulch Films: Highly Stretchable PLA Composites with Different Industrial Vegetable Waste
Source: ACS Appl Mater Interfaces. 2022 Oct 5;14(41):46920–31. doi: 10.1021/acsami.2c10965 (PMC9585519; doi:10.1021/acsami.2c10965)
Supplement: Supplementary file 1 — am2c10965_si_001.pdf [file am2c10965_si_001.pdf]

SUPPORTING INFORMATION

Biodegradable and bio-based mulch films: highly stretchable PLA composites with different industrial vegetable waste

Danila Merino[1, *]; Arkadiusz Zych[1]; Athanassia Athanassiou[1, *]

[1]Smart Materials Group, Istituto Italiano di Tecnologia, Via Morego, 30, Genoa, 16163 Italy

[*]Corresponding author: Dr. Danila Merino and Dr. Athanassia Athanassiou, Smart Materials Group, Italian Institute of Technology (IIT), Via Morego 30, 16163, Genoa, Italy. Tel: +39 010 28961. E-mail: danila.merino@iit.it; danila_m04@hotmail.com; athanassia.athanassiou@iit.it.

SI. *FTIR*

Table S1. The infrared spectroscopy wavenumber and their assignment for PLA, ESOME, PPLA and their composites with SS, TP and CS.

Sample	Wavenumber (cm^{-1})						
	C-H stretch	C=O stretch	C-H bend	C=O bend	C-O- stretch	Epoxide	C=C, N-H & C-N
**PLA**	2995, 2943 & 2849	1746	1450	1267	1179, 1127 & 1077	-	-
**ESOME**	3000, 2951 & 2856	1748	1436 & 1460	1246		825	
**PPLA**	2995, 2943 & 2856	1747	1450	1268	1179, 1127 & 1079	-	-
**PPLA+10SS**	2995, 2943 & 2856	1747	1450	1268	1179, 1127 & 1079	-	1696-1540
**PPLA+20SS**	2995, 2943 & 2855	1747	1450	1268	1179, 1127 & 1079	-	1696-1540
**PPLA+30SS**	2995, 2943 & 2852	1746	1450	1268	1179, 1127 & 1079	-	1696-1540
**PPLA+10TP**	2995, 2943 &2857	1747	1450	1268	1179, 1127 & 1079	-	-
**PPLA+20TP**	2995, 2925 &2854	1747	1450	1268	1179, 1127 & 1079	-	1696-1540
**PPLA+30TP**	2995, 2925 &2854	1745	1450	1268	1179, 1127 & 1079	-	1696-1540
**PPLA+10CS**	2995, 2925 & 2854	1746	1450	1268	1179, 1127 & 1079	-	1696-1540
**PPLA+20CS**	2995, 2925 & 2854	1746	1450	1268	1179, 1127 & 1079	-	1696-1540
**PPLA+30CS**	2995, 2925 & 2854	1742	1450	1268	1179, 1127 & 1079	-	1696-1540

SI. *Morphology of dried and milled vegetable waste*

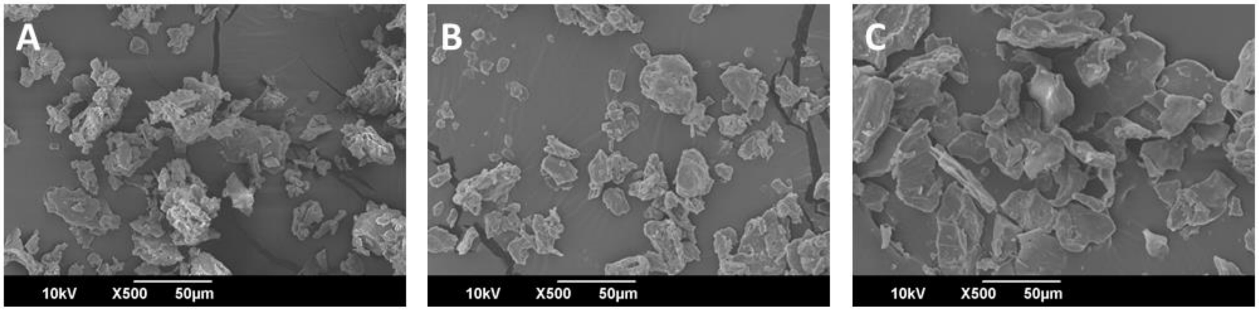

Figure S1 SEM micrographs of **A**: CS powder, **B**: SS powder, and **C**: TP powder.

__SI.__ Mechanical properties of PLA and PPLA

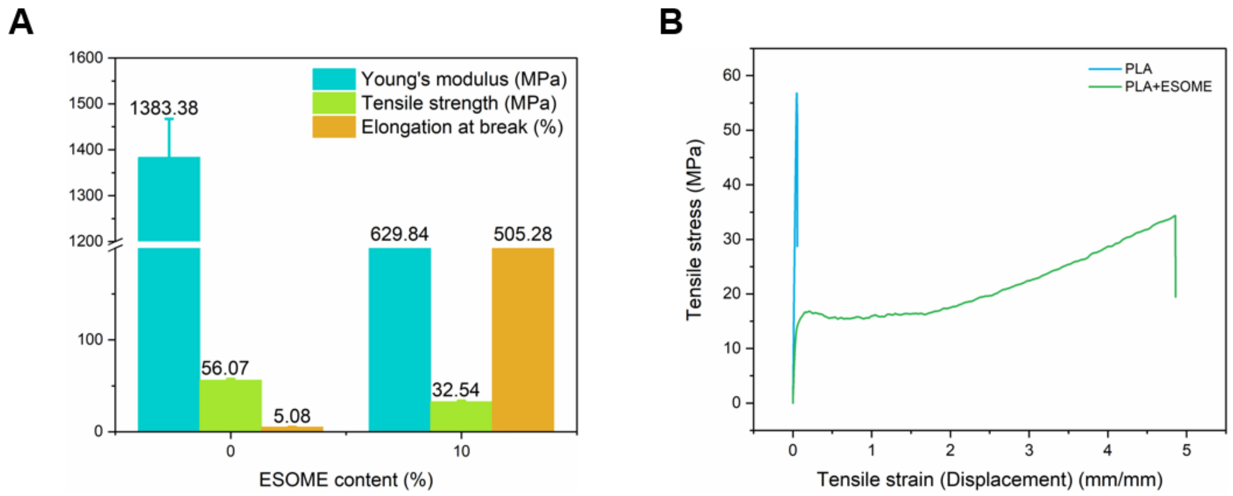

Figure S2 Changes in mechanical properties of PLA after plasticization with 10 wt.% of ESOME. **A**: Comparison between Young's modulus (MPa), Tensile strength (MPa) and Elongation at break (%) of PLA and PLA+10wt.% of ESOME. **B**: Tensile stress (MPa) vs Tensile strain (mm/mm) curves for PLA and PLA+10 wt.% ESOME.

SI. *SEM micrographs over the biodegradability test*

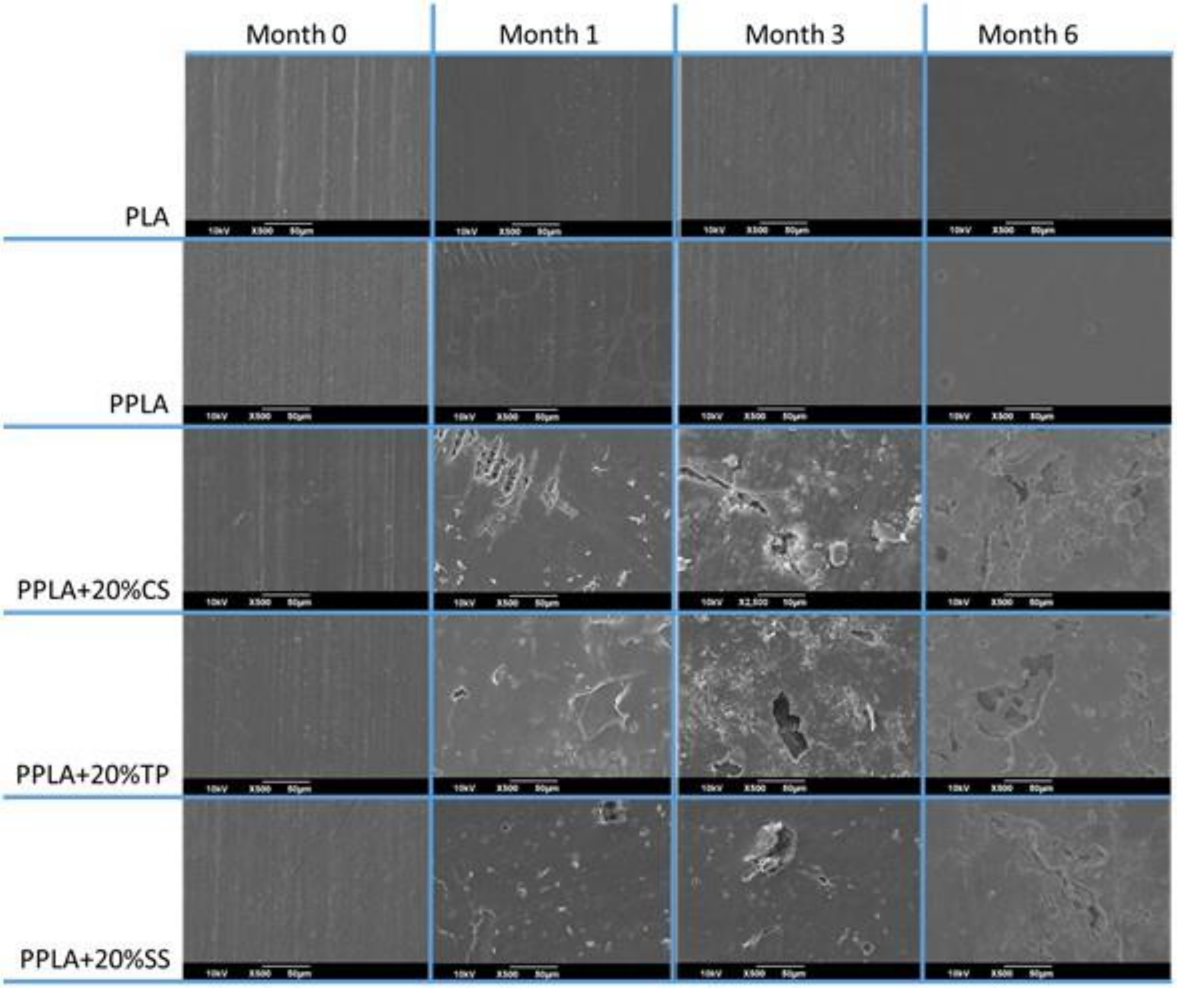

Figure S3 SEM micrographs of the surface of PLA, PPLA and PPLA composites with SS, TP and CS before (0 months) and after biodegradation in soil during 1, 3 or 6 months.

SI. *Molecular weights by GPC*

Table S2 Changes in PLA molecular weight after biodegradation in soil during 6 months.

Sample	Calculated by OMNISEC Mw (g/mol)	Calculated by OMNISEC Mn (g/mol)	Calculated by OMNISEC Đ	Calculated manually Mw (g/mol)	Calculated manually Mn (g/mol)	Calculated manually Đ
**PLA – month 0**	112 030	152 790	1.36	152 527	204 405	1.34
**PLA – month 6**	112 877	153 434	1.36	148 405	197 846	1.33
**PPLA – month 0**	100 367	141 609	1.41	152 002	202 536	1.33
**PPLA – month 6**	65 827	96 285	1.46	141 576	185 537	1.31
**PPLA – 20%SS month 0**	290 133	298 747	1.03	144721	189526	1.31
**PPLA – 20%SS month 6**	292 597	303 755	1.04	133 942	176 593	1.32

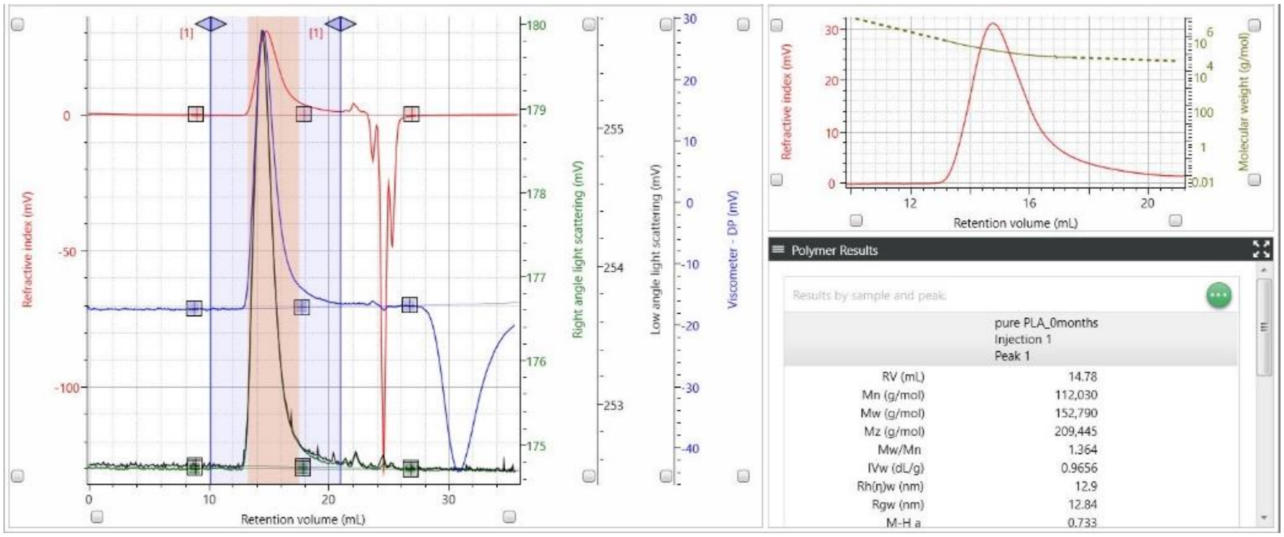

Figure S4 GPC curves and molecular weight calculation for PLA – month 0 sample.

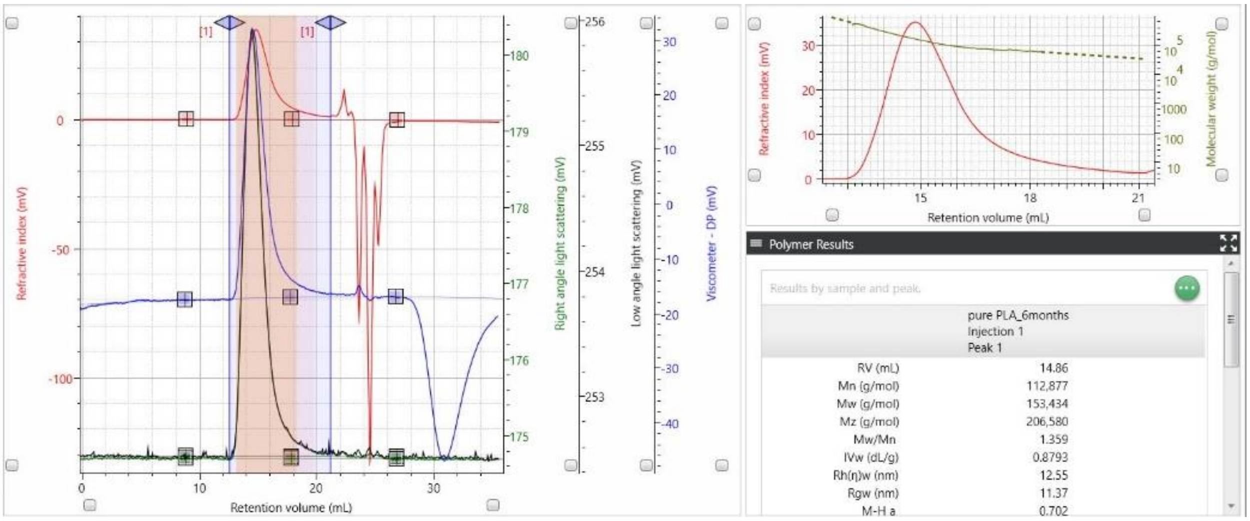

Figure S5 GPC curves and molecular weight calculation for PLA – month 6 sample.

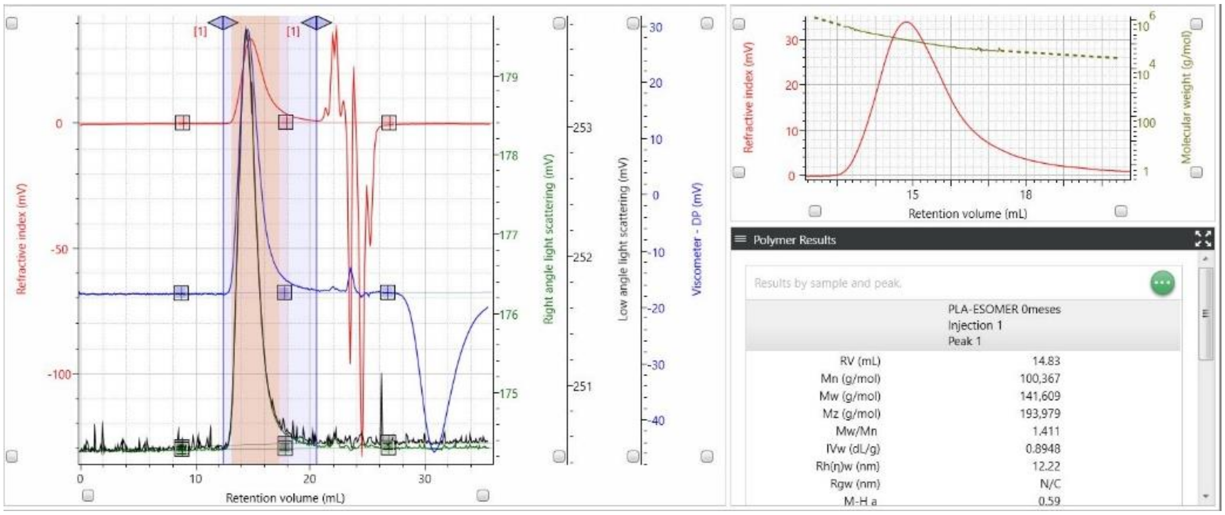

Figure S6 GPC curves and molecular weight calculation for PPLA – month 0 sample.

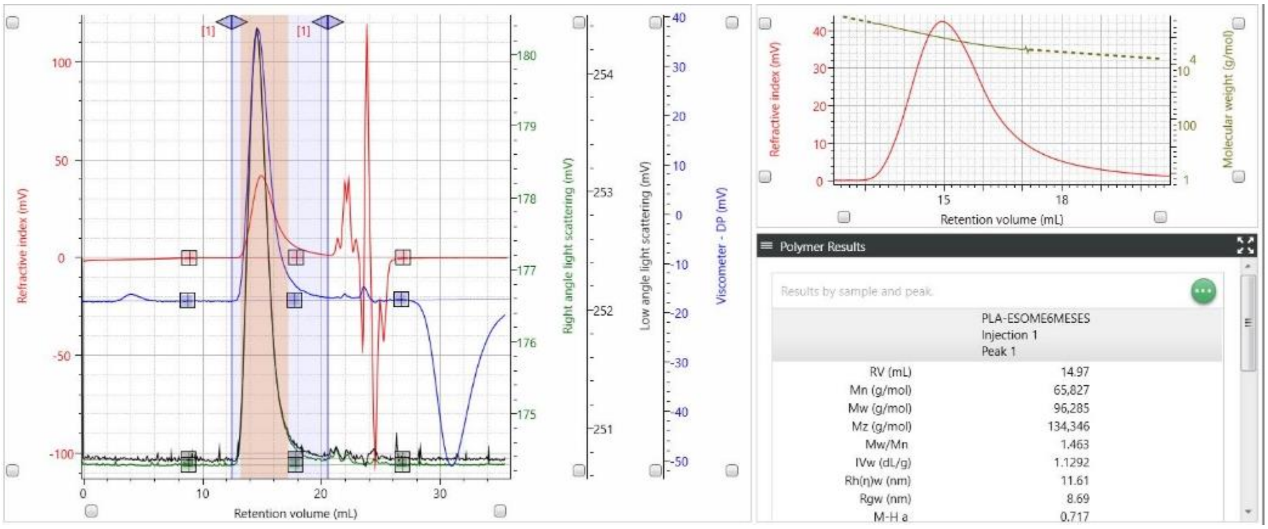

Figure S7 GPC curves and molecular weight calculation for PPLA – month 6 sample.

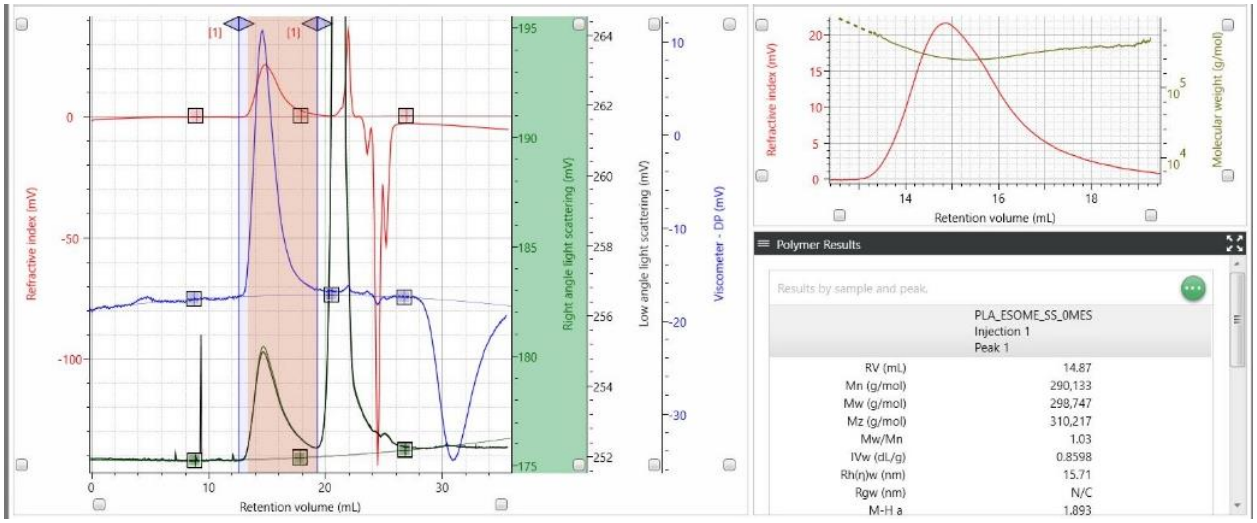

Figure S8 GPC curves and molecular weight calculation for PPLA – 20%SS month 0 sample.

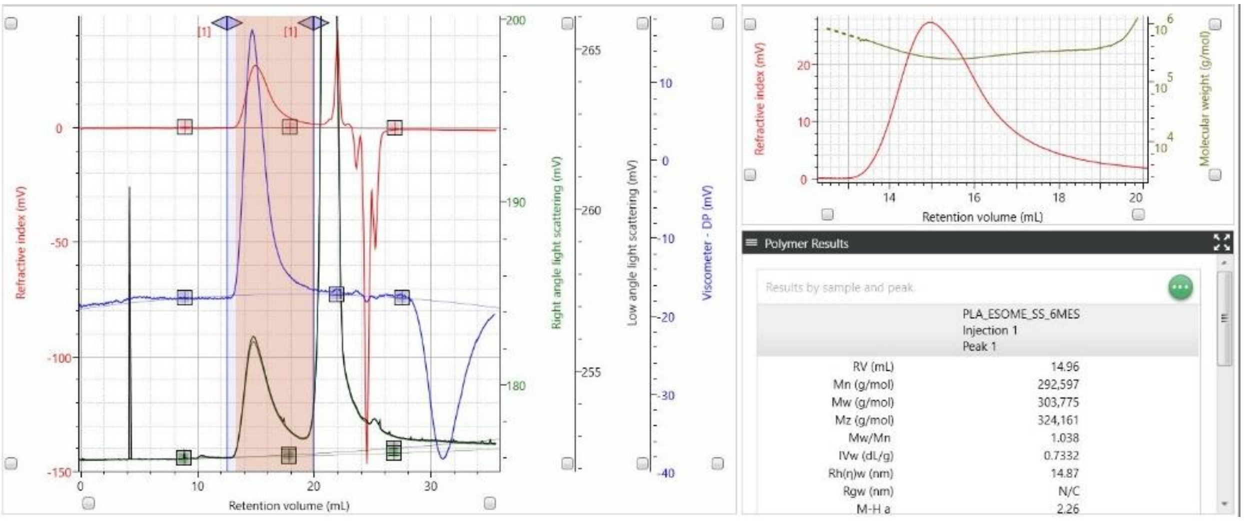

Figure S9 GPC curves and molecular weight calculation for PPLA – 20%SS month 6 sample.

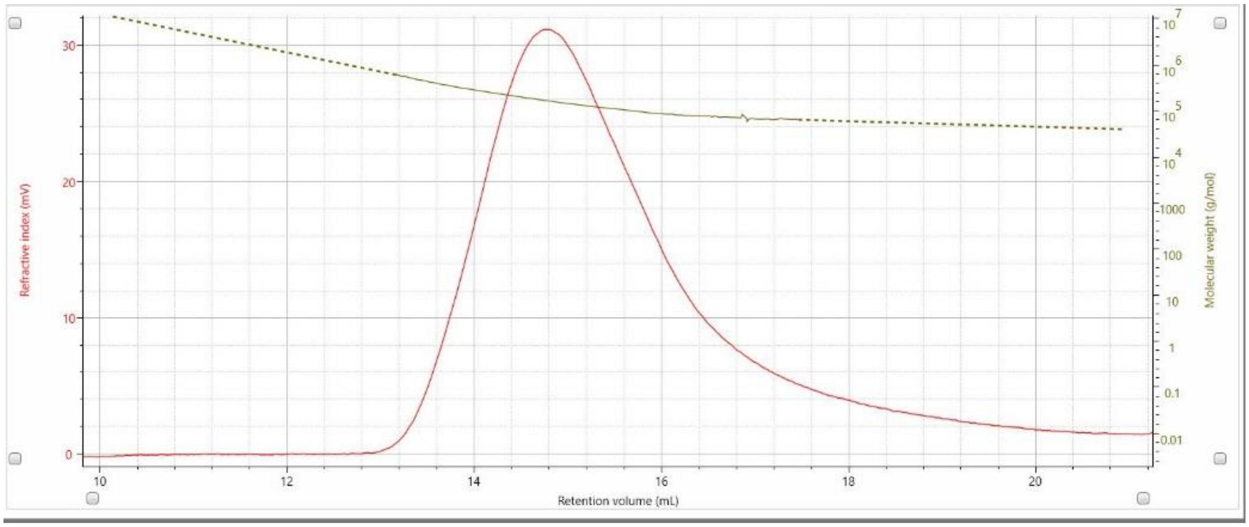

Figure S10 Relationship between the retention volume and the molecular weight established for pure PLA sample.
